# The Impact of Probiotic Supplementation on the Development of the Infant Gut Microbiota: An Exploratory Follow-Up of a Randomised Controlled Trial

**DOI:** 10.3390/microorganisms13050984

**Published:** 2025-04-25

**Authors:** Niall Coates, Daniel A. John, Sue Jordan, Melanie Storey, Catherine A. Thornton, Iveta Garaiova, Duolao Wang, Stephen J. Allen, Daryn R. Michael, Susan F. Plummer, Paul D. Facey

**Affiliations:** 1 Research & Development, Cultech Ltd., Port Talbot SA12 7BZ, UK; niallc@cultech.co.uk (N.C.); danielj@cultech.co.uk (D.A.J.); ivetag@cultech.co.uk (I.G.); darynm@cultech.co.uk (D.R.M.); suep@cultech.co.uk (S.F.P.); 2 Faculty of Medicine, Health and Life Science, Swansea University, Swansea SA2 8PP, UK; s.e.jordan@swansea.ac.uk (S.J.); m.storey@swansea.ac.uk (M.S.); c.a.thornton@swansea.ac.uk (C.A.T.); 3 Liverpool School of Tropical Medicine, Liverpool L3 5QA, UK; duolao.wang@lstmed.ac.uk (D.W.); stephen.allen@lstmed.ac.uk (S.J.A.); 4 Biomedical Sciences, Swansea University, Swansea SA2 8PP, UK

**Keywords:** infant gut microbiota development, multi-strain probiotic, atopy, DNA sequencing

## Abstract

Early-life establishment of the gut microbiota plays a role in lifelong health, with disruptions linked to heightened risks of metabolic and immune disorders. Probiotic supplementation may be used to modulate the infant gut microbiome to promote favourable development. Here, we evaluate how Lab4B probiotic supplementation shapes the development of the infant gut microbiome over the first 6 months. Faecal samples collected from infants enrolled in PROBAT (ISRCTN26287422), a randomised, double-blind, placebo-controlled trial, were analysed using culture-dependent and -independent (16S rDNA and metagenomic shotgun sequencing) techniques to examine the composition, diversity, and metabolic capabilities of the microbiome, as well as the abundance of antimicrobial resistance genes (ARGs). Probiotic supplementation encouraged the development of a microbiome with a distinct composition characterised by elevated abundances of *Bifidobacteriaceae* in the first 6 weeks (*p* = 0.006) and *Lactobacillaceae* throughout the first 6 months (*p* < 0.05 at every 6-week time point), accelerated microbial diversification, reduced abundance of beta-lactam- and cephalosporin-resistance genes, and differences in predicted metabolic capabilities at the start and end points. Supplementation of this neonatal population, which is at high risk of atopy, with the Lab4B probiotic significantly influenced the development of the infant gut microbiota during the first 6 months.

## 1. Introduction

The community of microorganisms that reside in the human gastrointestinal tract, known collectively as the human gut microbiota, is increasingly recognised for its critical role in overall health, influencing metabolic, immunological, and neurological functions throughout life [1,2,3]. The establishment of the gut microbiota plays a pivotal role in shaping how the immature immune system develops, with implications for health and quality of life during childhood and beyond [4,5,6]. Recent studies have proposed a “typical” pattern of microbiota developmental progression within the first year of life that supports the development of a healthy immune system [7,8,9,10]. This progression is characterised by the dominance of *Bifidobacterium* species, which utilise the human milk oligosaccharides (HMOs) in breastmilk [5,11], help establish a low abundance of pathobionts [8], and achieve a balanced rate of diversification that is neither delayed nor accelerated [9,12]. Practices such as caesarean delivery, feeding infant formula, and antibiotic exposure may disrupt this “typical” pattern [7,13,14] and are associated with higher incidences of immunological disorders, including allergies, atopy, and autoimmune diseases [9,12,15]. Given the potential lifelong implications of establishing a healthy gut microbiome during infancy, there is significant interest in exploring the use of interventions to support the development of the microbiota [2,16,17].

Probiotics are one possible intervention for the modulation of the developing infant gut microbiota. Probiotics are defined by the World Health Organisation as ‘live microorganisms which when administered in adequate amounts confer a health benefit on the host’ [18], and several studies report beneficial effects in lowering the incidences of atopy [16,19] and eczema [20], especially when given both prenatally to mothers and postnatally to infants [21]. However, studies have shown mixed results in terms of whether probiotic supplementation can alter the composition of the gut microbiota in infants born after 37 completed weeks of gestation [22].

The ‘Probiotics in the Prevention of Atopy in Infants and Children’ (PROBAT) study (ISRCTN26287422), was a large, randomised, double-blind, placebo-controlled trial that assessed the ability of the Lab4B probiotic to prevent the development of atopic conditions in a cohort of 454 newborns [23]. The study cohort predominantly comprised infants with an anticipated increased risk of developing atopy (those with a first-degree relative with clinically diagnosed asthma, eczema, or allergic rhinitis). The probiotic reduced the incidence of atopic eczema and protected against sensitisation to cow’s milk and egg proteins. Details of the study procedures are described elsewhere [24].

This exploratory follow-up study investigated differences in gut microbiome development between infants in the Lab4B probiotic and placebo groups. Faecal samples from a subset of the first 300 PROBAT trial participants were analysed using plate culture and DNA sequencing techniques.

## 2. Materials and Methods

### 2.1. Study Design and Sample Numbers

During the PROBAT trial, expectant mothers in the final month of pregnancy were randomly assigned (1:1) to receive a daily supplement of the Lab4B multi-strain probiotic or a matching placebo until delivery, with their babies receiving the same daily intervention until the age of 6 months; additional details can be found in the supplementary CONSORT flow diagram. Randomisation was performed by external commercial partners using a computer-generated random sequence without blocks [23]. Participants and researchers were blind to study-group allocation until data collection was completed and the databases were locked [23]. Ethical approval was granted in February 2004 by the South West Wales Research Ethics Committee on behalf of NHS Wales (Project Ref. HE09 COL 1002), and recruitment took place between May 2005 and October 2007 [25]. Although the inclusion criteria required that participants be mothers carrying a foetus with a first-degree relative clinically diagnosed with atopy, this criterion was not always met.

The probiotic daily dose of 1 × 10^10^ colony-forming units (CFU) comprised 6.25 × 10^9^ CFU *Ligilactobacillus salivarius* CUL61 (National Collection of Industrial, Food and Marine Bacteria (NCIMB 30211) and 1.25 × 10^9^ CFU each of *Lacticaseibacillus paracasei* CUL08 (NCIMB 30154), *Bifidobacterium animalis* subsp. *lactis* CUL34 (NCIMB 30172) and *Bifidobacterium bifidum* CUL20 (NCIMB 30153) on a maltodextrin base. The placebo comprised maltodextrin only and was identical in appearance to the probiotic. The intervention was taken orally by the mothers, and for the infants, the powder was either sprinkled into the infant’s open mouth or mixed with infant formula/expressed breast milk.

Carers of the infants were contacted by a research assistant at home and/or over the phone at 6, 12, 18, and 24 weeks to complete questionnaires that recorded information on common symptoms, compliance with the intervention (number of unused capsules), feeding method, visits to general practitioners, hospital admissions, medicines administered (including any antimicrobials), any adverse events, and the infant’s general health [24,25].

Carers were requested, but not obliged, to submit nappies containing their infants’ faecal samples at birth, 2 weeks, 6 weeks, 12 weeks, 18 weeks, and 6 months, but provision was sporadic. Nappies were collected by the carers, transferred to Anaerogen bags (Sigma-Aldrich, Gillingham, UK), anaerobically sealed, and stored in a refrigerator prior to transfer to the laboratory (typically within 3 days) for storage at −80 °C pending further analysis. 

For this follow-up analysis, antibiotic-treated infants were excluded, and faecal samples from which high-quality DNA was extracted were analysed; the final sample set included 105 samples from 46 infants in the placebo group and 113 samples from 54 infants in the probiotic group.

### 2.2. Faecal Viable Numbers

At the time of the original trial, faecal samples were enumerated by traditional plate counting within three months of their arrival at the laboratory, with selective agars used to quantify viable *Lactobacilli*, *Bifidobacteria*, *Enterobacter*, *Enterococci*, *Bacteroides*, *Staphylococci*, *Streptococci*, and *Clostridia*, as well as yeast, total aerobes, and total anaerobes, following the method of Madden et al. [26]. The detection limit was 5 log_10_ CFU/g faecal dry weight. Any faecal material not used for this testing was stored at −80 °C.

### 2.3. Genomic DNA Extraction and Quantification

In 2021, the faecal samples were removed from storage and genomic DNA was extracted. The samples were mechanically lysed using Matrix Lysing B tubes in conjunction with a FastPrep-24^TM^ bead beater (MP Biomedicals, Solon, OH, USA) for three cycles of 30 s at 5 m/s (with a 5-min interval between cycles). Genomic DNA was extracted from the lysed samples using the QIAamp Fast DNA stool kit (Qiagen, Manchester, UK), and DNA concentrations were measured using a Qubit^®^ 2.0 Fluorometer (Invitrogen, Waltham, MA, USA), with both procedures carried out in accordance with the manufacturer’s instructions. Prior to sequencing, the quality of extracted genomic DNA was assessed by running 20 ng of total isolated gDNA on a 0.7% agarose gel. Intact, non-sheared gDNA was evidenced by the appearance of discrete, high-molecular-weight bands on the gel. Isolated gDNA was diluted to 4 ng/µL in nuclease-free water.

### 2.4. Genomic Analysis

Samples that yielded high-quality DNA were categorised as follows by the age of the infant at the time of sampling: 0 to 6 weeks (T1), 7 to 12 weeks (T2), 13 to 18 weeks (T3) and 19 to 24 weeks (T4). A subset of these samples was analysed in more depth by metagenomic shotgun sequencing; this subset included 16 samples (8 placebo and 8 probiotic) from 16 infants up to 2 weeks old (Starting Point [SP]) and 18 samples (5 placebo and 13 probiotic) from 18 infants aged between 19 and 24 weeks (Endpoint [EP]).

### 2.5. Analysis of the Faecal Microbiota by 16S rDNA

#### 2.5.1. Amplicon Sequencing and Initial Processing

16S rDNA sequencing from the genomic DNA extracts was performed in accordance with a previously described method [27]. Briefly, sample libraries were prepared using a modified version of Illumina’s 16S Metagenomic Sequencing Library Preparation Protocol targeting the V1-V2 hypervariable region of the 16S rRNA gene [28]. Sample libraries were quantified using the NEBNext Library Quant Kit for Illumina (New England Biolabs, Hitchin, UK), and sequencing was performed on an Illumina MiSeq platform with the MiSeq Reagent Kit v3 (Illumina Inc., Saffron Walden, UK) using paired-end 2 × 300 bp chemistry. Raw sequencing data were processed following the dada2 (v1.34.0) [29] pipeline in R. Paired-end reads were filtered and trimmed to ensure all nucleotides had a Q score ≥ 30. Forward and reverse reads were merged and chimeric sequences removed. The resulting amplicon sequence variants (ASVs) were assigned taxonomy via alignment to the SILVA database v138 [30].

#### 2.5.2. Bacterial Taxonomic Analysis

The tax_glom function of the R package phyloseq (v1.50.0) [31] was used to collapse amplicon sequence variants (ASVs) to the level of family, genus or species, and counts were converted to relative abundance. The 10 most abundant bacterial families across all samples and all time points were identified by summing the relative abundance in all samples, and the remaining families (77) were combined into the ‘Other’ category. Relative abundance data were centre-log-ratio (CLR) transformed using the microbiome R package (v 1.28.0) [32] before statistical analysis.

#### 2.5.3. Differential Abundance

At each time point, differential genera were identified with Maaslin2 (v1.20) [33] using relative abundance data, controlling for the random effect of the participant and setting all covariates as fixed effects (genera present in 10% of samples were compared with the Compound Poisson Linear Model (CPLM) analysis method, default transformation and no normalisation). The false discovery rate (FDR) was controlled with the Benjamini–Hochberg method with a significance threshold of Q ≤ 0.05.

#### 2.5.4. Diversity Measures

Alpha and beta diversities were calculated with phyloseq. Alpha diversity was quantified from untransformed species counts with Shannon and Simpson diversity indices. Beta diversity was analysed by generating Bray–Curtis dissimilarity matrices from relative abundance data at each time point and visualised with non-metric multidimensional scaling (NMDS) plots.

#### 2.5.5. Analysis of Neonatal Community State Type (CST)

Partitioning around medoids (PAM) analysis (cluster R package; v2.17 [34]) was applied to a Jensen—Shannon divergence (JSD) matrix derived from genus-level relative abundance data for the T1 samples. The optimal number of clusters (community state types, CSTs) was determined by maximising the median sample silhouette width (Si) and optimising the ratio of positive to negative Si scores across clusters, with k ranging from 2 to the square root of the number of samples. Cluster medoids were visualised with the JSD matrix on a principal coordinates analysis (PCoA) plot using the “cmdscale” function (k = 2). Bacterial genera (relative abundance) and independent variables (study group and covariates) were assessed for their explanatory power on the ordination using the “envfit” function of the vegan R package (v2.6–8) [35]. The relationship between independent variables and each CST was assessed using CST membership as a dependent variable and following the approach outlined in Section 2.7.

#### 2.5.6. Microbial Networks

Data were split by study group at each time point, and group sample numbers were balanced (using the ‘sample’ function in R [base v4.4.2]) to avoid sample size-disparity bias when comparing networks [36]. The NetCoMi R package (v1.10) [37] with the SPRING method [38] was used to generate networks from the CLR-transformed relative abundances of genera present in at least 10% of samples. Each node (genus) in a network had a score calculated for the following metrics of centrality: degree (number of direct connections to other nodes), betweenness (frequency of appearing on the shortest path between nodes), closeness (proximity to other nodes) and eigenvector (influence on highly connected nodes). For each measure of centrality, the genera that scored in the top quartile of the network were considered ‘keystones’ and were compared between study groups at each time point. Network information was imported into Gephi (v0.10.1) [39] for visual styling and structured using the OpenOrd force-directed algorithm. Nodes were coloured by bacterial family, and their sizes were scaled by ‘hub score’, which is the sum of normalised values for degree, betweenness, closeness, and eigenvector centrality scores. Edge size represents the strength of an association between genera; green edges represent positive associations (mutualism), while red edges represent negative associations (antagonism).

### 2.6. Analysis of the Faecal Microbiota by Metagenomics

#### 2.6.1. Shotgun Sequencing and Initial Processing

Shotgun metagenomic sequencing of the genomic DNA extracts was performed using the Illumina NovaSeq 6000 platform (Novogene, Beijing, China) with 150-nucleotide long paired-end reads. Adaptor removal and quality control of raw reads was conducted by Novogene. Quality was confirmed using FASTQC (v0.12.0) [40], ensuring all base calls had a Q score of at least 30. Host-sequence decontamination was performed using Bowtie2 (v2.5.4) [41] (default parameters) against the GRCh38 genome.

#### 2.6.2. Microbial Profiling and Gene Prediction

The relative abundance of microbial taxa was determined by mapping reads to the CHOCOPhlAn database with MetaPhlAn4 (v4.1.1) [42] using default parameters. Reads were assembled into contigs using MEGAHIT (v1.2.8) [43], and open reading frames (ORFs) were predicted with Prodigal (v2.6.3) in metagenomic mode [44]. Redundant ORFs were clustered and consolidated into representative sequences using CD-HIT (v4.8.1) [45] with criteria of >95% identity and >90% coverage and were used to create a custom database. Metagenomic reads were aligned to the custom database using Bowtie2 with the ‘–very-sensitive-local’ parameters. Mapped and unmapped reads were separated, sorted, and indexed using SAMtools (v1.2) [46]. The number of reads mapped to each gene was calculated, and the counts were normalised to reads per kilobase per million mapped reads (RPKM).

#### 2.6.3. Annotation of Antibiotic-Resistance Genes, Mobile Genetic Elements and Metabolic Pathways

Identification of antibiotic-resistance genes (ARGs) was performed using the Resistance Gene Identifier (RGI, v6.0.3) provided by the Comprehensive Antibiotic Resistance Database (CARD, v 3.3.0 [47]). The abundance of mobile genetic elements (MGEs) was evaluated with ShortBred (v.0.9.4) [48] using a reference database of MGEs [49]. Metabolic-pathway profiling was performed using HUMAnN (v3.0) with the MetaCyc database (accessed on 2 August 2024 at https://metacyc.org/) [50] as a reference. Differential abundance analysis of pathways at each time point was performed in DESeq2 (v1.46.0) [51] after filtering for pathways present in at least 10% of samples. *p*-values were adjusted using the Benjamini–Hochberg correction.

### 2.7. Statistical Analysis

All statistical analyses of the 16S rDNA data, except for beta diversity and microbial-network comparisons, were conducted via generalised linear mixed models (GLMMs) with the glmmTMB R package (v1.1.10) [52]. Each model included the participant as a random effect, with study group, time point, and their interaction as fixed effects. Any of the following covariates were included as fixed effects if they significantly reduced the Akaike information criterion (AIC): delivery mode (vaginal or caesarean), number of weeks of breastfeeding, infant’s intervention compliance as an interaction with group, sex of infant, Townsend index score, presence of a sibling, Good’s coverage (for 16S rDNA data) and infant’s age in days (if separate models were required for each time point). Model details can be found in Supplementary Tables S3, S5, S9 and S11. To assess the significance of fixed effects, the emmeans R package (v1.10.3) [53] was used to calculate marginal means from model coefficients and perform contrasts with a Tukey’s adjustment to control the false discovery rate.

To analyse beta diversity, we first assessed the community dispersion (within-group variability) of each group at each time point using the betadisper and permutest functions [35]. If significant differences in community dispersion were detected between groups, we balanced group sizes using the sample function in R to minimise the risk of false positives arising when we compared community compositions using PERMANOVA (vegan) [35,54]. PERMANOVA tests included covariates as fixed effects if they accounted for a significant amount of variance. Within-group community dispersion between time points was compared using pairwise permutests, and community composition was assessed using pairwise PERMANOVA [55]. All PERMANOVAs were run with 999 permutations.

Significant differences in keystone taxa between group microbial networks were assessed using Jaccard’s dissimilarity index (NetCoMi package).

Between- and within-group comparisons of the abundance of ARGs and MGEs were assessed with the two-tailed Mann–Whitney U test.

For all statistical analyses, * represents differences between groups at a time point and # represents the difference within groups over time, with the comparative time point indicated proximally to the hashtag. Significance was considered and represented as follows: */# *p ≤* 0.05, **/## *p ≤* 0.01, ***/### *p ≤* 0.001.

## 3. Results

### 3.1. Characteristics of the Study Population

Table 1 provides a summary of the demographic, environmental, behavioural, and socio-economic characteristics of the population, stratified by group. Rates of health-related events during the 6-month study period for infants in this sub-study are presented in Supplementary Table S1.

### 3.2. Viable Microbial Numbers

**Figure 1:**
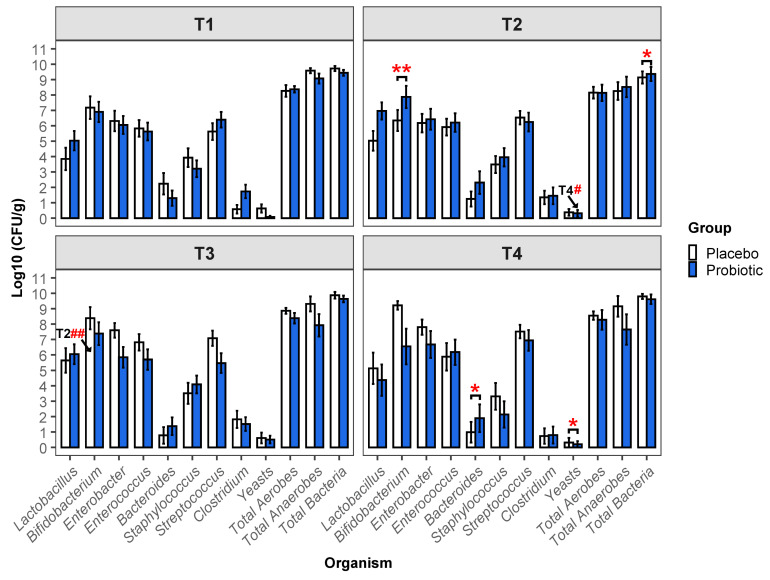
Data on viable organisms are presented as log_10_ CFU/g dry weight and represent mean ± SEM (sample/infant numbers; presented in Supplementary Data). Statistically significant differences between groups at each time point are indicated by asterisks (* *p* ≤ 0.05, ** *p* ≤ 0.01). Significant differences between time points within a group are indicated by hashtags (# *p* ≤ 0.05, ## *p* ≤ 0.01), with the comparative time point indicated proximally to the hashtag.

The viable counts for bacteria and yeast enumerated on selective media are presented in Figure 1 (detailed data in Supplementary Table S2). Between-group differences were observed at T2, when the probiotic group had both a higher abundance of *Bifidobacterium* spp. and higher total bacterial numbers (7.87 vs. 6.34 log_10_ CFU/g, *p =* 0.008 and 9.36 vs. 9.14 log_10_ CFU/g, *p =* 0.040, respectively). At T4, *Bacteroides* spp. numbers were higher in the probiotic group than in the placebo group (1.89 log_10_ CFU/g and 0.89 log_10_ CFU/g, *p =* 0.025). At T4, numbers of yeasts, although very low, were significantly higher in the placebo group than in the probiotic group (0.31 vs. 0.2 log_10_ CFU/g, *p =* 0.014, respectively).

Within-group differences in viable numbers indicated an increase in the number of *Bifidobacterium* spp. from T2 to T3 in the placebo group (6.34 vs. 8.38 log_10_ CFU/g, *p =* 0.002) and a decrease in the number of yeasts from T2 to T4 in the probiotic group (0.31 vs. 0.2 log_10_ CFU/g, *p =* 0.023).

### 3.3. 16S Analysis of Faecal Microbiota

#### 3.3.1. Relative and Differential Abundance of Bacterial Taxa

**Figure 2:**
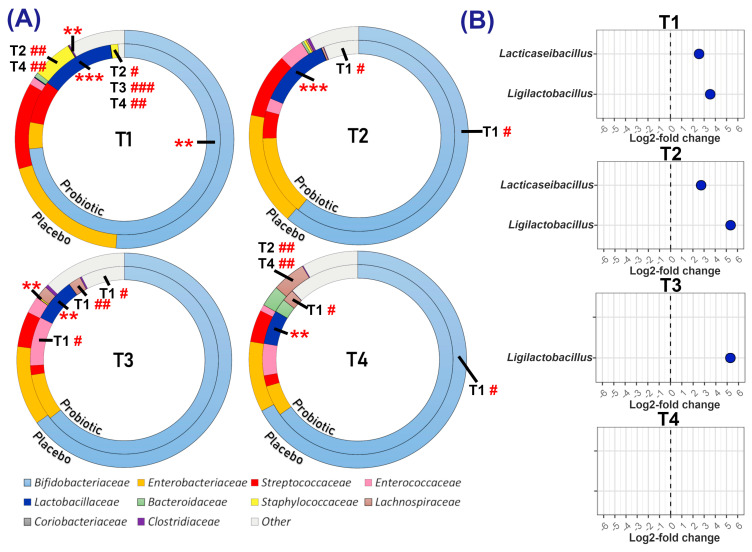
Comparison of bacterial abundance by family and genus. (**A**) Donut plots represent the median relative abundance of the 10 most abundant bacterial families, with all remaining families aggregated as ‘Other’. Statistically significant differences between groups at each time point are indicated by asterisks (** *p* ≤ 0.01, *** *p* ≤ 0.001). Significant differences between time points within a group are indicated by hashtags (# *p* ≤ 0.05, ## *p* ≤ 0.01, ### *p* ≤ 0.001), with the comparative time point indicated proximally to the hashtag. (**B**) Bacterial genera found to be significantly differentially abundant in the probiotic group relative to the placebo group.

The abundances of the 10 most prevalent bacterial families differed between the probiotic and placebo groups (Figure 2A, Supplementary Figure S1 and Table S4). The probiotic group was significantly enriched in the *Lactobacillaceae* family throughout the study and had a higher prevalence of the *Bifidobacteriaceae* at T1. The placebo group had higher abundances of the *Clostridiaceae* and the *Staphylococcaceae* at T1 and T3, respectively.

At T1, *Bifidobacteriaceae* represented >70% of the bacterial population in the probiotic group, compared to 51.2% in the placebo group; this value significantly increased in the placebo group by T4, reaching 67.17% (*p =* 0.006). T1 abundances of the *Staphylococcaceae* were 5.96% in the placebo group and 1.24% in the probiotic, but *Staphylococcaceae* were not detectable in either group at the end of the intervention period (*p =* 0.002 and 0.006, respectively). Both groups had an increase in *Lachnospiraceae* abundance from T1 to T4 (placebo: 0.09% to 4.98%; *p =* 0.024, probiotic: 0% to 2.07%; *p =* 0.005).

Between-group differential abundance analysis of all bacterial genera (Figure 2B) indicated significantly higher proportions of *Lacticaseibacillus* at T1 and T2 and *Ligilactobacillus* at T1, T2, and T3 in the probiotic group. Statistical modelling focused on the abundance of the supplemented probiotic organisms (Table 2) confirmed the differential-abundance analysis results, showing significant differences between groups for *Ligilactobacillus* and *Lacticaseibacillus* at T1, T2, and T3. Furthermore, the probiotic group was enriched in *B. bifidum* at all time points and in *B. animalis* at T2 and T3.

#### 3.3.2. Alpha and Beta Diversity

**Figure 3:**
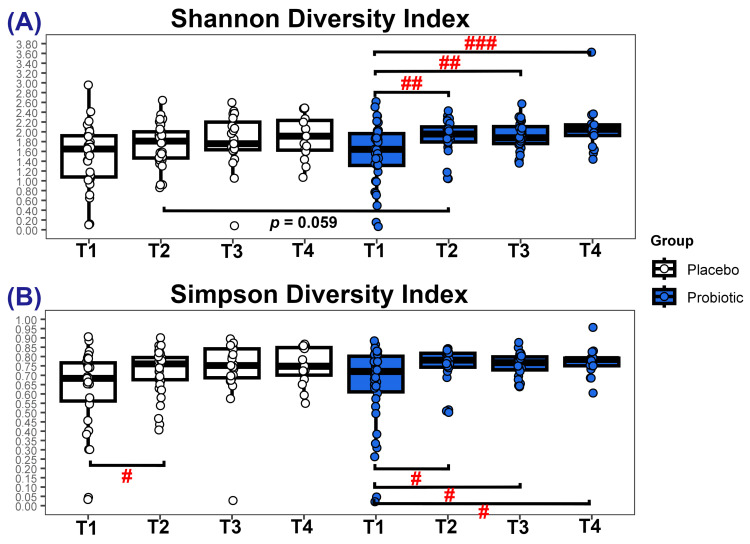
Alpha diversity. Points represent individual faecal samples. (**A**) Shannon and (**B**) Simpson alpha diversity indices. Values of *p*: # *p* ≤ 0.05, ## *p* ≤ 0.01, ### *p* ≤ 0.001.

There were no significant differences in alpha diversity between groups (Figure 3A,B), although at T2, there was a trend (*p* = 0.059) toward a higher Shannon diversity index for the probiotic cohort (median = 1.95 vs. 1.81). For the probiotic group, both Shannon and Simpson diversity indices increased significantly over time, whereas in the placebo cohort, Shannon diversity remained unchanged but Simpson diversity increased between T1 and T2.

**Figure 4:**
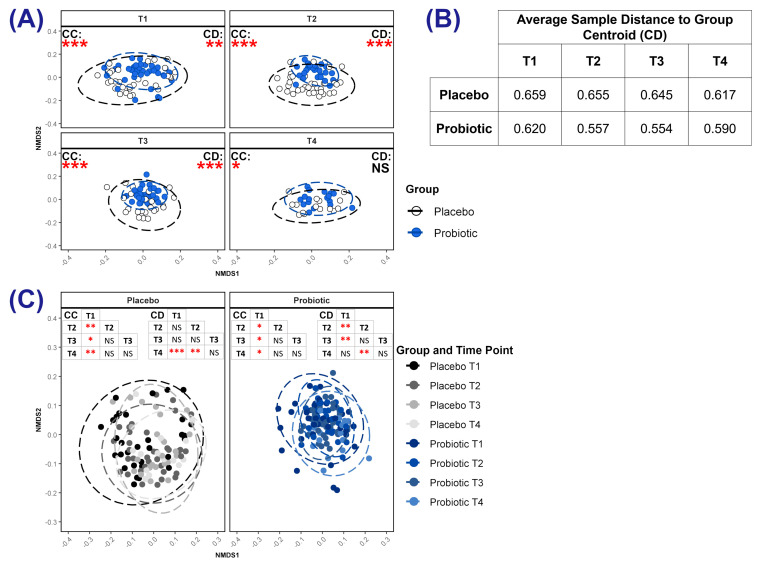
Beta diversity. Points represent individual faecal samples, and ellipses indicate 95% confidence intervals around centroids. CC (community composition) is a test of differences in ordination space of group/time-point centroids; CD (community dispersion) is a test of the variance of sample dispersion around the group/time-point centroid. (**A**) Comparison of beta diversity between groups at each time point; (**B**) values of community dispersion within groups at each time point; (**C**) comparison of beta diversity at each time point within groups. Values of *p*: * *p* ≤ 0.05, ** *p* ≤ 0.01, *** *p* ≤ 0.001.

Visualisation of beta diversity revealed that the probiotic and placebo group centroids occupied distinct positions in ordination space at each time point (Figure 4A and Supplementary Table S6), a finding indicative of differences in microbial community composition. There was lower variability in the community composition (community dispersion) of samples within the probiotic group than within the placebo group, as calculated from the distance of samples to the group’s centroid (*p =* 0.006 at T1, *p ≤* 0.001 at T2 and at T3, *p ≤* 0.001; Figure 4A,B and  Supplementary Table S7). Within each group, the community composition at T1 was distinct from those at other time points (Figure 4C and Supplementary Table S8). Additionally, community dispersion varied within groups between time points (Figure 4B,C and Supplementary Table S7). For both groups, community dispersion was highest at T1 and declined over time. For the placebo group, this reduction was only significant by T4 (average sample distance to centroid at T4 was 0.617 vs. 0.659 at T1; *p ≤* 0.001), whereas, for the probiotic group, the community dispersion at T2 was significantly lower than that at T1 (average sample distance to centroid was 0.620 at T1 but 0.557 at T2; *p =* 0.003) and remained lower until T4, when community dispersion was comparable to that at T1.

#### 3.3.3. Neonatal Community State Type (CST)

**Figure 5:**
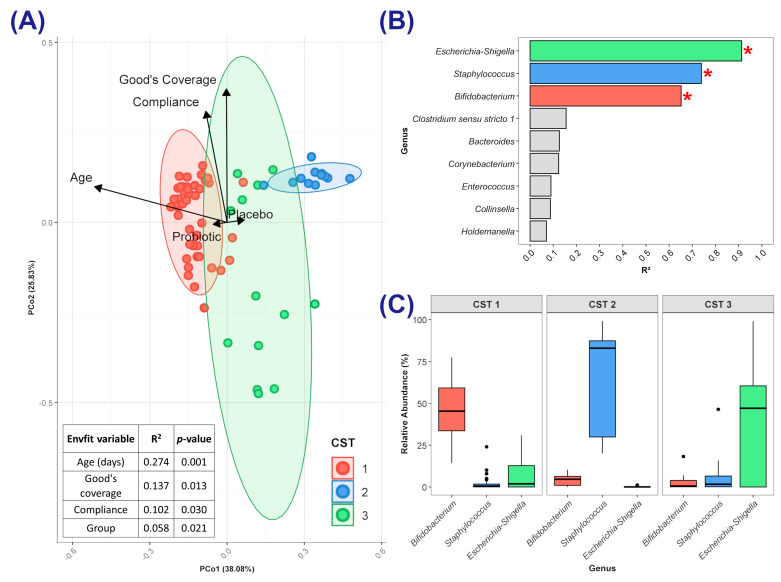
Analysis of community state type (CST) at Time Point 1. (**A**) Ordination plot of faecal microbiomes, showing samples coloured by CST with ellipses representing the 95% confidence interval around CST medoids. Vectors indicate significant environmental variables (*p* < 0.05), with length proportional to effect size. (**B**) Genera driving the ordination; colours indicate the CST where each genus dominates. Asterisks (*) denote significant variance explained (BH-adjusted *p* < 0.05). (**C**) Relative abundance (%) of significant genera for each CST, dots depict values larger than the upper quartile + 1.5 * IQR.

Analysis of the microbiotas in all the faecal samples at T1 identified three distinct community state types (CSTs), each characterised by predominance of a particular genus (Figure 5A–C). CST 1 was characterised by a high relative abundance of *Bifidobacterium* (median = 45.4%), CST 2 by *Staphylococcus* (82.9%), and CST 3 by *Escherichia-Shigella* (47.1%). The abundance of each of these genera explained a significant (after FDR adjustment) proportion of the variance in the ordination (Figure 5B): *Escherichia–Shigella* R^2^ = 0.91 (*p =* 0.046), *Staphylococcus* R^2^ = 0.74 (*p =* 0.046), *Bifidobacterium* R^2^ = 0.65 (*p =* 0.046). Some variance in microbial community composition was explained by the following independent variables (Figure 5A): infant’s age (R^2^ = 27.4%; *p <* 0.001), Good’s Coverage scores (R^2^ = 13.7%; *p =* 0.013), infant’s compliance with the intervention (R^2^ = 10.2%; *p =* 0.03), and whether the infant was in the probiotic or placebo group (R^2^ = 5.8%; *p =* 0.021). CST 1 included 44 samples (15 placebo and 29 probiotic; 63.77% of all T1 samples), whereas CST 2 and CST 3 comprised 12 (7 placebo and 5 probiotic; 17.4%) and 13 (9 placebo and 4 probiotic; 18.84%) of the T1 samples, respectively. 

Associations between independent variables and membership of each CST were evaluated (Supplementary Table S10). CST 1 was strongly associated with older infants (average age: 19 days, range: 1 to 42 days; adjusted odds ratio (AOR): 1.27 [1.06, 1.52] per one-day increase in age; *p =* 0.009) and trended toward association with the probiotic group (AOR: 5.69 [0.95, 33.42]; *p =* 0.057). CST 2 showed no significant association with independent variables, but CST 3 was associated with the following: (i) younger infants (average age: 5 days, range: 0 to 16 days; AOR: 0.86 [0.75, 0.98] per one-day increase in age; *p =* 0.028) (ii) higher rates of breastfeeding (AOR: 3.23 [1.27, 8.19] for each 20% increase in weeks breastfed; *p =* 0.041); (iii) lower compliance with the intervention (AOR: 0.43 [0.22, 0.83] for each 20% increase in compliance; *p =* 0.041).

#### 3.3.4. Microbial Networks and Keystone Taxa

**Figure 6:**
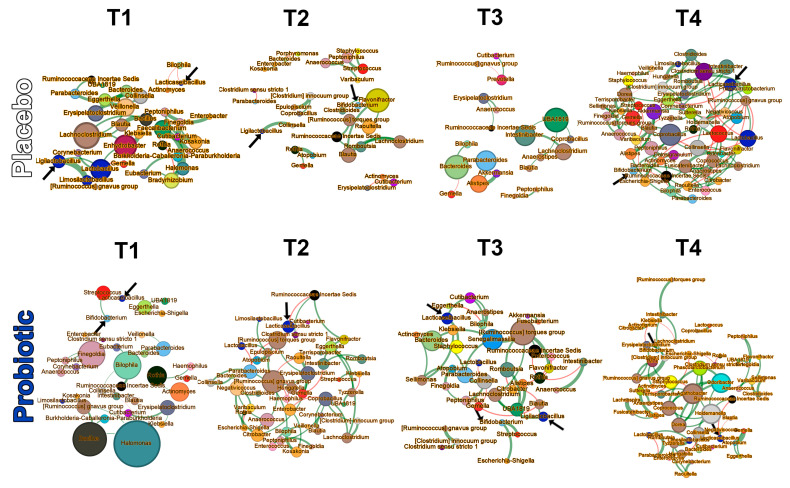
Genus-level bacterial networks; nodes represent bacterial genera coloured by family taxonomy and sized proportionally to their “hub score”. Green and red edges represent mutualistic and antagonistic relationships, respectively, between nodes. The strength of the relationship is proportional to the thickness of the edge. The bacterial genera that form part of the probiotic consortium are indicated with arrows.

Bacterial networks for the groups constructed at each time point (Figure 6 and Supplementary Figure S2) indicated that keystone taxa differed between groups at T1 (related to betweenness, closeness, and eigenvector centrality), at T2 (eigenvector centrality), at T3 (betweenness centrality), and at T4 (all measures; Table 3 and Supplementary Table S12). Descriptive and comparative metrics of the whole and of the most-connected component of each network are provided in the Supplementary Materials (Supplementary Table S13).

### 3.4. Metagenomic Analysis

#### 3.4.1. Abundance of Antibiotic-Resistance Genes (ARGs) and Mobile Genetic Elements (MGEs) 

**Figure 7:**
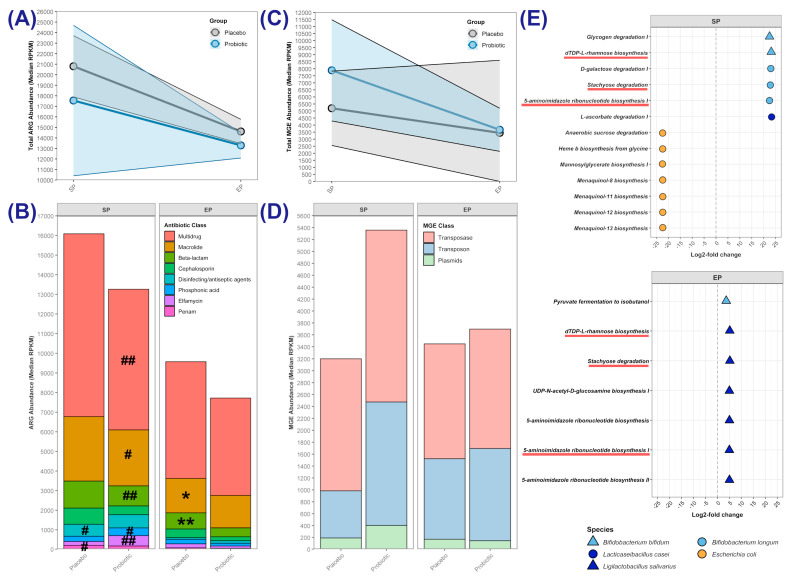
Resistome, mobilome, and differentially abundant metabolic pathways. Total abundance of antibiotic-resistance genes (ARGs) at the starting point (SP) and endpoint (EP); (**A**) total and (**B**) class medians. (**C**) Total abundance of mobile genetic elements (MGEs). (**D**) MGE class median abundances. Statistically significant differences between groups are indicated by asterisks (* *p* ≤ 0.05, ** *p* ≤ 0.01), whereas significant differences within a group are indicated by hashtags (# *p* ≤ 0.05, ## *p* ≤ 0.01), with the comparative time point indicated proximally to the hashtag. (**E**) Significantly differentially abundant metabolic pathways at the SP and EP. Log2-fold changes in the pathway abundance in the probiotic group relative to the abundance in the placebo group. Points are coloured by the bacterial genus from which the pathway originates. Species provided in the probiotic supplement are represented by triangles. Underlined pathways are differentially abundant at both time points.

No differences in total ARG abundance were observed between the groups (Figure 7A, Table 4 and Supplementary Table S14), but at EP in the placebo group, the genes associated with resistance to beta-lactams and cephalosporins were more abundant (*p =* 0.035 and 0.007, respectively).

Within the probiotic group, between SP and EP, there were decreases in the abundances of genes associated with resistance to beta-lactams (*p =* 0.010), elfamycin (*p =* 0.003), macrolides (*p =* 0.013), and phosphonic acid (*p =* 0.037), as well as a decrease in the abundance of genes associated with multidrug resistance (*p =* 0.008). In the placebo group, there were significant decreases in the abundances of genes associated with resistance to disinfecting/antiseptic agents (*p =* 0.011) and penams (*p =* 0.045).

Analysis of the mobilome (Figure 7C,D, and Table 5) revealed no significant differences between groups at either time point.

#### 3.4.2. Differentially Abundant Metabolic Pathways

Differential analysis of the abundance of metabolic pathways (Figure 7E and Supplementary Table S15) revealed that at the SP, the probiotic group was significantly enriched in the ‘glycogen degradation I’ and ‘dTDP-L-rhamnose biosynthesis’ pathways derived from *Bifidobacterium bifidum*, the ‘D-galactose degradation I’, ‘stachyose degradation’ and ‘5-aminoimidazole ribonucleotide biosynthesis I’ pathways from *Bifidobacterium longum*, and a ‘L-ascorbate degradation I’ pathway derived from *Lacticaseibacillus casei.* In contrast, the placebo group had a higher SP abundance of pathways derived from *Escherichia coli*, namely ‘anaerobic sucrose degradation’, ‘heme b biosynthesis from glycine’, and ‘mannosylglycerate biosynthesis I’, as well as four menaquinol biosynthesis pathways. At the EP, the probiotic group showed enrichment in ‘pyruvate fermentation to isobutanol’ from *Bifidobacterium bifidum* and several *Ligilactobacillus salivarius* pathways, specifically, ‘dTDP-L-rhamnose biosynthesis’, ‘stachyose degradation’, and ‘UDP-N-acetyl-D-glucosamine biosynthesis I’, as well as three ‘5-aminoimidazole ribonucleotide biosynthesis’ pathways. The ‘dTDP-L-rhamnose biosynthesis’, ‘stachyose degradation’, and ‘5-aminoimidazole ribonucleotide biosynthesis I’ pathways were enriched at both the SP and the EP, though their microbial sources differed between time points.

## 4. Discussion

In this subset of infants from the PROBAT study [23], we observed differences in gut microbiota development between those receiving the Lab4B probiotic and those receiving the placebo.

The microbiota composition and maturation from birth for the infants in the placebo group are comparable with those observed in other full-term infants [7,8,56,57], with *Bifidobacteriaceae* as the most abundant family (51%) at T1, followed in abundance by *Enterobacteriaceae* (19%) and *Streptococcaceae* (13%). In the probiotic group, *Bifidobacteriaceae* constituted 73% of the T1 microbiota and was followed in abundance by *Lactobacillaceae* (12.5%; largely *Lacticaseibacillus* and *Ligilactobacillus*), and *Streptococcaceae* (7%). For both groups, the abundance of *Staphylococcaceae* was highest at T1 and declined over time, and this has been seen in other studies [7,56]. As infants aged, the presence of bacterial families linked to a more mature microbiota—such as *Lachnospiraceae*, *Bacteroidaceae*, and *Ruminococcaceae*—increased in both groups, consistent with previous findings [8,13,14,58].

The probiotic group exhibited an age-related microbial diversification that has been seen in healthy infants [12], with temporal increases in the richness and evenness of species (Shannon index) alongside a reduction in the domination by a few groups (Simpson index). In contrast, the placebo group displayed no sustained increase in alpha diversity, potentially reflecting the high-atopy-risk population recruited for this study. Delays in microbiota diversification have been associated with the development of paediatric allergies [12], and it has been proposed that a ‘microbiota age’ that lags behind the infant’s chronological age could serve as a biomarker for predicting a predisposition to food allergies [59]. The infants in the placebo group of the PROBAT trial were found to have a higher incidence of food allergy [23], and this may be related to the observed composition of their microbiota.

Beta diversity analysis showed that the composition of infants’ gut microbiotas at T1 were disparate from those at later time points, and early differences in composition have been previously reported [7,56]. Probiotic supplementation appeared to support the development of a distinct enterotype, with between-group differences in community composition at all time points and lower variance between microbiotas (community dispersion) for the probiotic group at T1, T2, and T3.

We observed that the gut microbiomes of infants at T1 were most effectively partitioned into three distinct community state types (CSTs), each defined by the dominance of a specific genus, as reported by Shao et al. [15]. At T1, the infant’s age at the time of sampling was the primary driver of microbial differences, with older infants more likely to have a gut microbiome dominated by bifidobacteria (CST 1) and younger infants enriched in *Escherichia–Shigella* (CST 3). It has been found that the neonatal gut microbiome is initially dominated by *Enterobacteriaceae*, with a transition towards a *Bifidobacterium*-rich community as the neonatal gut matures and becomes increasingly anaerobic [7,13]. There are indications that the probiotic supported such a transition, with CST 1 showing a near-significant association with the probiotic group. The placebo group appeared to align more closely with CST 3.

The bifidobacteria species present in the Lab4B probiotic (*B. animalis* and *B. bifidum*) were more abundant in the infants in the probiotic group. Infants with a high abundance of bifidobacteria in their gut microbiome have been found to experience better health outcomes compared to those with lower levels; the benefits observed include a reduced risk of childhood obesity and metabolic disorders [5,11], enhanced resistance to pathogen colonisation [15], and a more tolerant immune system [60], which may contribute to the lower incidence of atopic diseases and cow’s milk allergy [12]. Bifidobacteria also play an important role in the maturation of the butyrogenic microbiota by cross-feeding acetate to support the establishment of butyrate-producing bacteria such as *Faecalibacterium prausnitzii*, *Agathobacter rectalis*, and species of *Anaerostipes*, *Eubacterium*, and *Roseburia* [61]. Microbially derived butyrate is known to support the integrity of the gut epithelium and reduce the risk of immune-mediated diseases, including food allergies [62,63].

In the probiotic group, *Lacticaseibacillus* spp. and *Ligilactobacillus* spp. (components of the probiotic) were detectable throughout the study. Members of the *Lactobacillaceae* family are typically enriched in vaginally-delivered and breastfed infants compared with caesarean-delivered or formula fed infants [5]. These bacteria are believed to contribute to positive health outcomes via production of metabolites that lower gastrointestinal pH and may inhibit enteric pathogens [5,64].

There were no between-group structural differences in the genus-level microbial networks, but keystone genera differed between the two networks at all time points. Keystone (hub) organisms have a large influence on the structure and function of ecological networks but are not necessarily the most abundant organisms [65]. They exert their influence through (i) a high number of direct connections to other organisms (degree centrality), (ii) frequent appearances on the shortest paths between pairs of organisms (betweenness centrality), (iii) being in close proximity to many other organisms (closeness centrality), or (iv) exertion of influence over other highly connected organisms within the network (eigenvector centrality). None of the Lab4B probiotic organisms was identified specifically as a keystone organism.

Analysis of the gut resistome revealed no between-group differences in the overall abundance of antibiotic-resistance genes (ARGs), but there were indications of a age/time-related decrease in abundance within both groups, as seen in previous studies [66,67,68]. Genes related to multidrug resistance were the most abundant in both groups, as found by Pärnänen et al. [49] and Casaburi et al. [69], and their abundance within the probiotic group decreased significantly between SP and EP. By EP, probiotic supplementation reduced the abundance of ARGs related to beta-lactams and cephalosporin in particular; these antibiotics are used as first-line treatments for serious infections in infants [70]. The abundance of mobile genetic elements was similar between the two groups throughout, suggesting that probiotic supplementation did not impact horizontal ARG transfer. Transposase genes constituted the largest fraction of mobile genetic elements in the gut metagenomes of our cohort, as has been reported for other infant populations [49].

Differential metabolic pathway analysis revealed that at the SP, the gut microbiomes of the probiotic group exhibited an enrichment of metabolic pathways related to carbohydrate metabolism, including galactose, glycogen, and stachyose degradation. The enrichment of these pathways may be a reflection of the extensive repertoire of carbohydrate-metabolising genes possessed by the bifidobacteria [71], whose abundance was higher in the probiotic group at T1. Enhanced galactose-degradation pathways may support an increased capacity for the gut microbiomes of the probiotic group to utilise galactose from breastmilk. The ‘stachyose degradation’ pathway, which was enriched in the probiotic group and attributed to *B. longum* at the SP and *L. salivarius* at the EP, enables the metabolism of stachyose (a prebiotic oligosaccharide) into acetate or propionate, which reduces intestinal pH and thereby can inhibit pathogen colonisation [72]. Metabolism of stachyose by the gut microbiota has been found to relieve inflammation [73] and reduce constipation in murine models [74]. Interestingly, Allen et al. [25] reported a reduced incidence of constipation in the infants in the Lab4B group. Peptidoglycan-synthesis pathways were also more abundant in the probiotic group, including ‘dTDP-L-rhamnose biosynthesis’ at both time points and ‘UDP-N-acetyl-D-glucosamine biosynthesis I’ at the EP. Peptidoglycan, a major component of bacterial cell walls, plays a critical role in shaping the developing innate immune system and influencing immune homeostasis [75]. Additionally, pathways for 5-aminoimidazole ribonucleotide biosynthesis were enriched at both SP and EP, potentially enhancing the synthesis of ribonucleotides—essential nutrients for infant gut and immune development that are primarily derived from breastmilk but are also synthesised by the gut microbiota [76,77,78]. The placebo group showed enrichment of metabolic pathways derived from *E. coli* at the SP, including several menaquinol (vitamin K2)-biosynthesis pathways. Vitamin K2 is primarily produced by gut bacteria and is vital for bone, cardiovascular, neural, and joint health, although its increased presence in faeces does not necessarily correlate with higher plasma levels [79]. Interestingly, the enrichment of ‘dTDP-L-rhamnose biosynthesis’ and D-galactose degradation I’ pathways, along with depletion of the ‘heme b biosynthesis from glycine’ pathway in the probiotic group, aligned with findings from a meta-analysis studying the probiotic supplementation of preterm infants [80].

A strength of this study was that both viable microbial numbers and molecular analysis were used to assess the development of the intestinal microbiome during early infancy with and without probiotic supplementation. Our analysis was limited to bacteria and did not consider the potential impact of probiotic supplementation on the gut mycobiome and virome. The inconsistency in the frequency of faecal-sample provision represented a weakness in this work that was addressed as far as possible through the statistical-analysis methodology applied. Additionally, the limited size of the cohort available for faecal analysis prevented the analysis of any correlations with the primary/secondary trial outcomes. Another observation is that our population subset had higher intervention compliance than the wider cohort, suggesting that carers who provided faecal samples were more likely to follow the recommended dosing protocol.

## 5. Conclusions

In summary, supplementation with the Lab4B probiotic influenced the development of the infant gut microbiome. The microbiomes of the infants in the probiotic group had an increased abundance of *Bifidobacteriaceae* and *Lactobacillaceae*, an accelerated diversification, lower levels of some antimicrobial-resistance genes, and predicted additional functional capabilities when compared with those of the control infants. Future longitudinal studies throughout infancy and beyond should be considered to gain a greater understanding of the longer-term impacts of probiotic supplementation during neonatal development.

## Figures and Tables

**Table 1:** Characteristics of the Study Population.

Variable	Placebo n = 46	Probiotic n = 54
Adherence to intervention in the first 6 weeks (mean ± SD)	74.3 ± 31.2%	66.4 ± 32.2%
Adherence to intervention over 6 months (mean ± SD)	71.39 ± 30.7%	66.25 ± 26.2%
Caesarean section	41.3%	35.2%
Female	45.7%	42.6%
Median birth weight in kg (IQR)	3.44 (0.66)	3.49 (0.77)
Sibling in household	43.5%	46.3%
Some breastfeeding	82.6%	77.8%
Breastfeeding (median no. weeks in 6 months (IQR))	7.5 (23)	5.5 (23)
Townsend score (median (min–max))	533 (89–1794)	795 (61–1891)
Townsend quintile 1	21.7%	18.5%
Townsend quintile 2	23.9%	16.7%
Townsend quintile 3	19.6%	25.9%
Townsend quintile 4	13.0%	24.1%
Townsend quintile 5	21.7%	14.8%
Number of infants with a first-degree relative with diagnosed atopy	84.8%	87.0%

**Table 2:** Relative abundance of supplemented organisms.

	T1	T2	T3	T4
	*B. animalis* Mean Relative Abundance ± SD (%)			
Placebo	0.00 ± 0.00	0.59 ± 2.00	0.42 ± 1.14	0.62 ± 2.41
Probiotic	0.49 ± 1.16	0.85 ± 1.31	0.50 ± 0.97	0.18 ± 0.30
*p*-value	0.998	0.001	0.043	0.331
	*B. bifidum* Mean Relative Abundance ± SD (%)			
Placebo	0.09 ± 0.27	0.18 ± 0.69	0.44 ± 0.91	0.19 ± 0.43
Probiotic	0.57 ± 0.83	0.86 ± 0.63	0.70 ± 0.44	0.70 ± 0.64
*p*-value	<0.001	<0.001	0.004	0.013
	*Lacticaseibacillus* Mean Relative Abundance ± SD (%)			
Placebo	1.01 ± 3.44	0.84 ± 2.61	2.71 ± 6.27	2.90 ± 6.57
Probiotic	6.83 ± 11.71	6.25 ± 7.66	4.02 ± 6.27	3.70 ± 7.31
*p*-value	<0.001	<0.001	0.031	0.206
	*Ligilactobacillus* Mean Relative Abundance ± SD (%)			
Placebo	0.18 ± 0.74	0.11 ± 0.66	1.36 ± 6.51	0.00 ± 0.00
Probiotic	6.04 ± 14.03	3.87 ± 5.72	3.36 ± 5.73	2.65 ± 3.86
*p*-value	<0.001	<0.001	<0.001	1.000
	Number of samples/infants			
Placebo	31/25	36/32	23/22	15/13
Probiotic	38/30	26/22	32/26	17/15

**Table 3:** Top five taxa for centrality measures, with different keystones between groups.

Time Point	Centrality Measure (*p*-Value)	Group (No. Samples/ Infants)	Network Size (No. Genera)	Keystones (Top 5)				
				Rank 1	Rank 2	Rank 3	Rank 4	Rank 5
T1	Betweenness (0.044)	Placebo (31/25)	40	*Faecalibacterium*	*Bradyrhizobium*	*Lachnoclostridium*	*Lactobacillus*	*Enhydrobacter*
		Probiotic (31/25)	29	*Halomonas*	*Bacillus*	*Bilophila*	*Finegoldia*	[*Ruminococcus*] *gnavus* group
T1	Closeness (0.002)	Placebo (31/25)	40	*Lachnoclostridium*	*Enhydrobacter*	*Lactobacillus*	*Faecalibacterium*	*Veillonella*
		Probiotic (31/25)	29	*Halomonas*	*Bacillus*	[*Ruminococcus*] *gnavus* group	*Bilophila*	*Finegoldia*
T1	Eigenvector (0.002)	Placebo (31/25)	40	*Lachnoclostridium*	*Enhydrobacter*	*Bacillus*	*Blautia*	*Bradyrhizobium*
		Probiotic (31/25)	29	*Bacillus*	*Halomonas*	[*Ruminococcus*] *gnavus* group	*Bilophila*	*Finegoldia*
T2	Eigenvector (0.026)	Placebo (26/25)	43	*Flavonifractor*	*Blautia*	*Raoultella*	*Lachnoclostridium*	*Peptoniphilus*
		Probiotic (26/21)	45	[*Ruminococcus*] *gnavus* group	[*Ruminococcus*] *torques* group	*Erysipelatoclostridium*	*Anaerococcus*	*Citrobacter*
T3	Betweenness (0.033)	Placebo (23/22)	40	*Bacteroides*	*UBA1819*	*Intestinibacter*	*Parabacteroides*	*Alistipes*
		Probiotic (23/20)	39	[*Ruminococcus*] *torques* group	*Bacteroides*	*Senegalimassilia*	*Staphylococcus*	*Collinsella*
T4	Degree (0.004)	Placebo (15/13)	48	*Fusicatenibacter*	*Clostridium sensu stricto 1*	*Coprobacillus*	*Lactococcus*	*Lactobacillus*
		Probiotic (15/13)	57	*Holdemanella*	*Agathobacter*	*Dorea*	*Odoribacter*	*Phascolarctobacterium*
T4	Betweenness (<0.001)	Placebo (15/13)	48	*Coprobacillus*	*Gemella*	*Dorea*	*Collinsella*	*Lactococcus*
		Probiotic (15/13)	57	*Agathobacter*	*Staphylococcus*	*Holdemanella*	*Odoribacter*	*Collinsella*
T4	Closeness (0.004)	Placebo (15/13)	48	*Coprobacillus*	*Lactobacillus*	*Lactococcus*	*Clostridium sensu stricto 1*	*Collinsella*
		Probiotic (15/13)	57	*Agathobacter*	*Holdemanella*	*Dorea*	*Odoribacter*	*Phascolarctobacterium*
T4	Eigenvector (<0.001)	Placebo (15/13)	48	*Lactobacillus*	*Clostridium sensu stricto 1*	*Coprobacillus*	*Lacticaseibacillus*	*Eggerthella*
		Probiotic (15/13)	57	*Dorea*	*Odoribacter*	*Phascolarctobacterium*	*Coprococcus*	*Holdemanella*

**Table 4:** Antibiotic-resistance genes showing significant within- or between-group changes.

	Within Placebo Group			Within Probiotic Group			Between Group Comparison	
	Median Abundance			Median Abundance				
	SP	EP	*p*-Value	SP	EP	*p*-Value	*p*-Value	
Antibiotic Class								
*Total*	20,801.05	14,610.85	0.065	17,550.55	13,291.35	0.121	0.798	0.566
*Multidrug*	9312.27	5949.31	0.222	7162.09	4964.88	0.008	0.959	0.703
*Macrolide*	3285.32	1755.77	0.065	2852.80	1655.09	0.013	0.505	1.000
*Beta-lactam*	1376.59	824.35	0.171	1022.68	460.44	0.010	0.645	0.035
*Cephalosporin*	827.68	448.33	0.524	444.36	201.70	0.121	0.083	0.007
*Disinfecting/antiseptic agents*	618.95	87.95	0.011	678.31	134.43	0.104	0.798	0.059
*Phosphonic acid*	268.09	230.47	0.724	397.25	142.55	0.037	0.878	0.566
*Elfamycin*	206.26	208.86	0.833	536.86	114.71	0.003	0.161	0.336
*Penam*	190.43	66.87	0.045	163.54	46.72	0.076	1.000	0.924
Number of infants	8	5		8	13			

**Table 5:** Abundance of mobile genetic elements.

	Within Placebo Group			Within Probiotic Group			Between Group Comparison	
	Median Abundance			Median Abundance				
	SP	EP	*p*-Value	SP	EP	*p*-Value	*p*-Value	
Mobile Genetic Elements								
*Total*	5194.99	3447.77	0.943	7888.70	3665.54	0.456	0.645	0.849
*Integron*	0.00	0.00	0.268	0.00	0.00	0.287	-	0.879
*Plasmids*	190.31	167.93	0.941	402.95	143.02	0.634	0.957	0.766
*Transposase*	2216.19	1925.02	0.622	2882.01	2001.04	0.972	0.721	0.633
*Transposon*	793.86	1354.81	0.509	2072.63	1552.11	0.856	0.873	1.000
Number of infants	8	5		8	13			

## Data Availability

The data that support the findings of this study are available from the corresponding author upon reasonable request. Sequence data generated during the current study have been submitted to the European Molecular Biology Laboratory (EMBL) nucleotide sequence database (https://www.ebi.ac.uk/ena [accessed on 9 December 2024]) under accession number PRJEB82926.

## Supplementary Materials

The following supporting information can be downloaded at: https://www.mdpi.com/article/10.3390/microorganisms13050984/s1, Figure S1: Individual Sample Relative Abundance of the Most Abundant Bacterial Families. The 10 most abundant bacterial families are presented with the remaining families aggregated into an “Other” category. Multiple samples from the same infant within a time point are designated with an A or B after the participant number; Figure S2: title; Family Level Bacterial Networks; Nodes represent bacterial family coloured by taxonomy and sized proportionally to the “hub score”. Green and red edges represent mutualistic and antagonistic relationships, respectively, between nodes. The strength of the relationship is proportional to the thickness of the edge. The bacterial families that form part of the probiotic consortium are indicated with arrows; Table S1: Sensitivity to common allergens and rates of atopic eczema and health-related observations at the end of the 6-month intervention period; Table S2: Viable Counts (Log_10_ CFU/g) at Each Time Point; Table S3: Viable Plate Culture—Statistical Models and Covariates; Table S4: Relative Abundance of the Most Prevalent Bacterial Families at Each Time Point; Table S5: Bacterial Family—Statistical Models and Covariates; Table S6: Beta Diversity—Community Composition—Between Group Comparisons; Table S7: Beta Diversity—Community Dispersion; Table S8: Beta Diversity—Community Composition—Within Group Comparison; Table S9: Diversity Measures—Statistical Models and Covariates; Table S10: Neonatal Community State Type Independent Variables (*p* < 0.1); Table S11: Neonatal Community State Type—Statistical Models and Covariates; Table S12: Full list of Taxa for Centrality Measures with Different Keystones between Groups; Table S13: Microbial Network Metrics; Table S14: Abundance of All Antibiotic Resistance Gene Classes Identified in this Study; Table S15: MetaCyc pathway IDs.

## Abbreviations

The following abbreviations are used in this manuscript:

Abbreviation	Expansion
PROBAT	Probiotics in the Prevention of Atopy in Infants and Children
SP	starting point
EP	endpoint
CST	community state type
IQR	interquartile range
AOR	adjusted odds ratio
CFU	colony-forming units
gDNA	genomic DNA
ASV	amplicon sequence variant
16S rDNA	16S ribosomal DNA
CLR	centre-log-ratio
ARG	antibiotic-resistance gene
MGE	mobile genetic element
ORF	open reading frame
RPKM	reads per kilobase per million mapped reads
PCoA	principal coordinates analysis
NMDS	non-metric multidimensional scaling
JSD	Jensen–Shannon Divergence
GLMM	generalised linear mixed model
AIC	Akaike information criterion
FDR	false discovery rate
DADA2	Divisive Amplicon Denoising Algorithm 2
NCIMB	National Collection of Industrial, Food and Marine Bacteria
CARD	Comprehensive Antibiotic Resistance Database
RGI	Resistance Gene Identifier
DESeq2	Differential Expression Sequencing version 2
HUMAnN	The HMP Unified Metabolic Analysis Network
MetaCyc	metabolic pathway database
CHOCOPhlAn	A pan-genome database used with MetaPhlAn
NetCoMi	Network Construction and Comparison for Microbiome Data
