# Supplementary material for: The Impact of Probiotic Supplementation on the Development of the Infant Gut Microbiota: An Exploratory Follow-Up of a Randomised Controlled Trial
Source: Microorganisms. 2025 Apr 25;13(5):984. doi: 10.3390/microorganisms13050984 (PMC12114409; doi:10.3390/microorganisms13050984)
Supplement: Supplementary file 1 [file microorganisms-13-00984-s001.zip › Supplementary material - Review Update.pdf]

The Impact of Probiotic Supplementation on Infant Gut Microbiota Development: An Exploratory Follow-Up of a Randomised Controlled Trial

Supplementary Material

Supplementary Table S1. Sensitivity to common allergens and rates of atopic eczema and health-related observations at the end of the 6-month intervention period

As recorded in Allen et al., 2014 [23]

Abbreviations: SPT, skin prick test; OR, odds ratio; CI, confidence interval

	**Probiotic (n=54)**	**Placebo (n=46)**	**OR (95% CI)**	***p*-value**
**SPT† positive, n (%)**	2/48 (4.17%)	5/41 (12.20%)	0.31 (0.06 to 1.63)	0.241
· Cows' milk	0/46 (0.00%)	2/41 (4.88%)	-	0.219
· Egg	2/46 (4.35%)	3/41 (7.32%)	0.58 (0.10 to 2.96)	0.663
· House dust mite	0/48 (0.00%)	0/41 (0.00%)	-	>0.999
· Cat	0/48 (0.00%)	2/41 (4.88%)	-	0.209
· Grass	1/48 (2.08%)	0/41 (0.00%)	-	>0.999
**Atopic eczema††, n (%)**	1/51 (1.96%)	5/45 (11.11%)	0.16 (0.01 to 1.28)	**0.095**
**Common symptoms, n (%)**				
· Regurgitation	50/54 (92.59%)	44/46 (95.65%)	0.57 (0.10 to 2.55)	0.684
· Colic	33/54 (61.11%)	33/46 (71.74%)	0.62 (0.27 to 1.39)	0.295
· Diarrhoea	23/54 (42.59%)	22/46 (47.83%)	0.81 (0.35 to 1.83)	0.688
· Constipation	18/54 (33.33%)	28/46 (60.87%)	0.32 (0.14 to 0.74)	**0.009**
· High temperature	13/54 (24.07%)	19/46 (41.30%)	0.45 (0.20 to 1.04)	**0.086**

†valid if diameter of wheal for the positive control was ≥1 mm than diameter for the negative control; SPT positive if diameter of wheal for antigen was ≥3 mm than diameter for negative control

†† defined as a positive SCORAD score and at least one positive SPT

Values of *p* determined using the Fisher Exact test in GraphPad Prism version 10.2.0

Supplementary Table S2: Viable Counts (Log10 CFU/g) at Each Time Point

	PLACEBO GROUP										PROBIOTIC GROUP										PLACEBO VS PROBIOTIC			
	Mean (SEM)				P-value (within group comparison):						Mean (SEM)				P-value (within group comparison):						P-value (between group comparison):			
	T1	T2	T3	T4	T1 vs T2	T1 vs T3	T1 vs T4	T2 vs T3	T2 vs T4	T3 vs T4	T1	T2	T3	T4	T1 vs T2	T1 vs T3	T1 vs T4	T2 vs T3	T2 vs T4	T3 vs T4	T1	T2	T3	T4
No. samples/infants:	28/23	36/32	23/22	15/13							37/29	26/22	31/26	16/14										
**Organism**																								
Lactobacillus	3.85 (0.73)	5.02 (0.64)	5.64 (0.79)	5.13 (1.01)	0.950	0.989	0.889	0.995	0.987	0.959	5.03 (0.63)	6.97 (0.55)	6.05 (0.64)	4.37 (1.02)	0.271	0.443	0.960	0.981	0.756	0.905	0.805	0.375	0.458	0.653
Bifidobacterium	7.17 (0.73)	6.34 (0.68)	8.38 (0.72)	9.21 (0.28)	0.755	0.052	0.753	**0.002**	0.221	0.623	6.9 (0.66)	7.87 (0.72)	7.38 (0.74)	6.55 (1.15)	0.361	0.600	0.998	0.964	0.403	0.638	0.858	**0.008**	0.246	0.398
Enterobacter	6.31 (0.67)	6.18 (0.6)	7.59 (0.47)	7.8 (0.49)	0.892	1.000	0.989	0.932	0.992	0.995	6.05 (0.58)	6.42 (0.68)	5.84 (0.66)	6.67 (0.87)	0.852	0.989	0.913	0.670	1.000	0.800	0.270	0.704	0.234	0.993
Enterococcus	5.83 (0.54)	5.91 (0.54)	6.82 (0.53)	5.88 (0.89)	0.505	0.788	0.822	0.986	0.997	1.000	5.63 (0.57)	6.21 (0.6)	5.7 (0.66)	6.18 (0.82)	0.920	0.921	0.724	0.591	0.433	0.968	0.324	0.383	0.476	0.386
Bacteroides	2.23 (0.69)	1.24 (0.49)	0.78 (0.54)	0.98 (0.67)	0.809	0.617	0.074	0.968	0.221	0.424	1.3 (0.5)	2.31 (0.73)	1.38 (0.58)	1.89 (0.9)	0.332	0.924	0.638	0.721	0.993	0.920	0.150	0.250	0.616	**0.025**
Staphylococcus	3.92 (0.61)	3.48 (0.55)	3.51 (0.68)	3.31 (0.87)	0.987	0.982	0.974	1.000	0.998	0.999	3.21 (0.55)	3.95 (0.59)	4.09 (0.57)	2.14 (0.85)	0.985	0.981	0.999	0.865	0.973	0.998	0.904	0.870	0.609	0.708
Streptococcus	5.63 (0.54)	6.53 (0.44)	7.08 (0.49)	7.51 (0.44)	0.584	0.245	0.741	0.853	1.000	0.911	6.39 (0.51)	6.25 (0.61)	5.47 (0.64)	6.94 (0.67)	0.981	0.994	0.999	0.999	0.997	1.000	0.137	0.963	0.524	0.875
Clostridium	0.58 (0.28)	1.35 (0.44)	1.82 (0.56)	0.73 (0.5)	0.123	0.304	0.842	0.921	0.668	0.858	1.73 (0.44)	1.45 (0.54)	1.51 (0.44)	0.8 (0.55)	0.613	0.999	0.878	0.686	0.301	0.845	0.088	0.439	0.811	0.921
Yeasts	0.62 (0.27)	0.37 (0.21)	0.61 (0.35)	0.31 (0.31)	0.558	0.870	0.872	0.280	0.284	1.000	0.07 (0.07)	0.31 (0.22)	0.51 (0.25)	0.2 (0.2)	0.321	0.729	0.994	0.399	**0.023**	0.249	0.481	0.225	0.292	**0.014**
Total aerobes	8.27 (0.38)	8.15 (0.38)	8.86 (0.2)	8.55 (0.28)	0.981	0.983	0.859	0.854	0.946	0.644	8.37 (0.21)	8.14 (0.53)	8.38 (0.34)	8.27 (0.63)	0.503	0.998	0.774	0.352	0.998	0.685	0.551	0.276	0.280	0.303
Total anaerobes	9.58 (0.17)	8.26 (0.57)	9.31 (0.48)	9.15 (0.67)	0.896	0.527	1.000	0.138	0.936	0.671	9.07 (0.32)	8.52 (0.66)	7.92 (0.73)	7.65 (0.99)	0.688	0.683	0.863	1.000	0.339	0.347	0.978	0.083	0.778	0.540
Total bacteria	9.72 (0.17)	9.14 (0.4)	9.87 (0.22)	9.79 (0.16)	0.782	0.722	0.976	0.159	0.985	0.552	9.44 (0.19)	9.36 (0.45)	9.63 (0.22)	9.61 (0.31)	0.412	0.758	0.942	0.924	0.905	0.998	0.753	**0.040**	0.653	0.547

Supplementary Table S3: Viable Plate Culture - Statistical Models and Covariates.

**Dependent Variable (CFU/g)**	**Minimal Fixed Effects**	**Additional Significant Fixed Effects**	**Model**
Lactobacillus	Group * Time point	Sibling	GLMM (tweedie family + log link)
Bifidobacterium	Group * Time point		GLMM (tweedie family + log link)
Enterobacter	Group * Time point	Breastfeeding	GLMM (tweedie family + log link)
Enterococcus	Group * Time point	Breastfeeding + Sibling	GLMM (tweedie family + log link)
Bacteroides	Group * Time point		GLMM (tweedie family + log link)
Staphylococcus	Group * Time point		GLMM (tweedie family + log link)
Streptococcus	Group * Time point	Breastfeeding	GLMM (tweedie family + log link)
Clostridium	Group * Time point		GLMM (tweedie family + log link)
Yeasts	Group * Time point	Breastfeeding	GLMM (tweedie family + log link)
Total aerobes	Group * Time point		GLMM (tweedie family + log link)
Total anaerobes	Group * Time point		GLMM (tweedie family + log link)
Total bacteria	Group * Time point		GLMM (tweedie family + log link)

Supplementary Table S4: Relative Abundance of the Most Prevalent Bacterial Families at Each Time Point

	PLACEBO GROUP										PROBIOTIC GROUP										PLACEBO VS PROBIOTIC			
	Median relative abundance (%):				P-value (within group comparison):						Median relative abundance (%):				P-value (within group comparison):						P-value (between group comparison):			
	T1	T2	T3	T4	T1 vs T2	T1 vs T3	T1 vs T4	T2 vs T3	T2 vs T4	T3 vs T4	T1	T2	T3	T4	T1 vs T2	T1 vs T3	T1 vs T4	T2 vs T3	T2 vs T4	T3 vs T4	T1	T2	T3	T4
No. samples/infants:	31/25	36/32	23/22	15/13							38/30	26/22	32/26	17/15										
**Bacterial Family**																								
Bifidobacteriaceae	51.25	61.25	65.09	67.17	**0.002**	0.151	**0.006**	0.647	0.978	0.540	73.40	60.89	64.44	65.20	0.271	0.280	0.657	1.000	0.979	0.989	**0.009**	0.439	0.076	0.891
Enterobacteriaceae	19.38	16.79	11.95	10.47	0.215	0.862	0.999	0.756	0.493	0.946	4.48	13.68	8.05	5.25	0.558	0.544	1.000	1.000	0.681	0.681	0.897	0.483	0.801	0.801
Streptococcaceae	12.81	9.56	5.34	4.78	0.244	0.990	0.998	0.503	0.565	1.000	7.21	4.60	1.58	1.85	0.961	0.793	0.809	0.552	0.607	0.999	0.436	0.584	0.682	0.729
Enterococcaceae	1.11	3.64	2.52	0.97	1.000	1.000	0.993	1.000	0.992	0.996	0.01	2.22	8.43	5.39	0.440	**0.012**	0.205	0.477	0.937	0.915	0.104	0.899	0.193	0.508
Lactobacillaceae	0.15	0.03	0.05	0.00	1.000	0.999	0.909	0.971	0.935	0.809	12.51	12.19	7.54	5.78	0.936	1.000	0.932	0.921	0.730	0.952	**0.000**	**0.000**	**0.004**	**0.009**
Bacteroidaceae	0.71	0.36	0.02	3.[illegible]3	1.000	0.965	0.981	0.965	0.975	0.862	0.08	0.00	0.00	2.34	0.995	1.000	0.656	0.998	0.586	0.647	0.568	0.457	0.912	0.852
Staphylococcaceae	5.96	0.35	0.19	0.00	**0.006**	0.058	**0.002**	0.984	0.385	0.289	1.24	0.05	0.00	0.00	**0.011**	**0.000**	**0.006**	0.446	0.804	0.985	0.151	0.152	**0.002**	0.689
Lachnospiraceae	0.09	0.25	1.77	4.98	0.975	0.506	**0.024**	0.697	**0.039**	0.418	0.00	0.34	2.18	2.07	0.270	**0.005**	**0.005**	0.502	0.393	0.980	0.165	0.997	0.782	0.377
Coriobacteriaceae	0.00	0.00	0.00	0.00	0.971	0.825	0.987	0.963	1.000	0.973	0.00	0.00	0.00	0.00	1.000	0.983	0.981	0.991	0.981	0.908	0.507	0.845	0.602	0.610
Clostridiaceae	0.18	0.39	0.49	0.[illegible]3	1.000	0.976	0.869	0.953	0.820	0.976	0.00	0.11	0.29	0.06	0.185	0.148	0.666	1.000	0.935	0.924	**0.004**	0.283	0.616	0.615
Other	8.35	7.39	12.56	8.36	0.182	0.544	0.589	0.951	0.989	0.999	1.08	5.92	7.50	12.05	**0.024**	**0.023**	0.283	0.998	0.903	0.940	0.731	0.217	0.134	0.554

Supplementary Table S5: Bacterial Family - Statistical Models and Covariates.

Dependent Variable (Centre Log Ratio Relative Abundance)	Minimal Fixed Effects	Additional Significant Fixed Effects	Model
Bifidobacteriaceae	Group * Time point		GLMM (tweedie family + log link)
Enterobacteriaceae	Group * Time point	Good's coverage	GLMM (tweedie family + log link)
Streptococcaceae	Group * Time point		GLMM (tweedie family + log link)
Enterococcaceae	Group * Time point		GLMM (tweedie family + log link)
Lactobacillaceae	Group * Time point		GLMM (tweedie family + log link)
Bacteroidaceae	Group * Time point	Birth mode	GLMM (tweedie family + log link)
Staphylococcaceae	Group * Time point		GLMM (tweedie family + log link)
Lachnospiraceae	Group * Time point	Breastfeeding	GLMM (tweedie family + log link)
Coriobacteriaceae	Group * Time point	Birth mode + Breastfeeding	GLMM (tweedie family + log link)
Clostridiaceae	Group * Time point		GLMM (tweedie family + log link)
Other	Group * Time point		GLMM (tweedie family + log link)

Supplementary Table S6: Beta Diversity – Community Composition - Between Group Comparisons

Community Composition - Between Group Comparison				
Time point	T1	T2	T3	T4
No. Samples/infants:				
Placebo	31/25	26/23	23/22	15/13
Probiotic	31/27	26/22	23/19	15/14
Group effect (R2)	0.036	0.086	0.078	0.059
Group (P-value)	≤0.001	≤0.001	≤0.001	0.015

Supplementary Table S7: Beta Diversity – Community Dispersion

PLACEBO GROUP

	Average Group Distance to Centroid				P-value (within group comparison):					
	T1	T2	T3	T4	T1 vs T2	T1 vs T3	T1 vs T4	T2 vs T3	T2 vs T4	T3 vs T4
No. samples/ infants:	31/25	36/32	23/22	15/13						
	0.659	0.655	0.645	0.617	0.617	0.171	**0.001**	0.303	**0.002**	0.067

PROBIOTIC GROUP

	Average Group Distance to Centroid				P-value (within group comparison):					
	T1	T2	T3	T4	T1 vs T2	T1 vs T3	T1 vs T4	T2 vs T3	T2 vs T4	T3 vs T4
No. samples/ infants:	38/30	26/22	32/26	17/15						
	0.620	0.557	0.554	0.590	**0.003**	**0.002**	0.157	0.856	**0.002**	0.113

PLACEBO VS PROBIOTIC

	P-value (between group comparison):			
	T1	T2	T3	T4
	**0.006**	**0.001**	**0.001**	0.226

Supplementary Table S8: Beta Diversity – Community Composition - Within Group Comparison

	Community Composition - Within Group Comparison												
	T1 vs T2	T1 vs T3	T1 vs T4	T2 vs T3	T2 vs T4	T3 vs T4		T1 vs T2	T1 vs T3	T1 vs T4	T2 vs T3	T2 vs T4	T3 vs T4
	Placebo							Probiotic					
**Variance explained by time point (R2)**	0.028	0.029	0.043	0.012	0.020	0.020		0.029	0.037	0.030	0.019	0.033	0.018
**Significance of time point (adjusted P-value [BH])**	**0.006**	**0.024**	**0.006**	0.919	0.678	0.919		**0.030**	**0.006**	**0.038**	0.390	0.159	0.656

Supplementary Table S9: Diversity Measures - Statistical Models and Covariates.

**Dependent Variable**	**Minimal Fixed Effects**	**Additional Significant Fixed Effects**	**Model**
Shannon's diversity scores	Group * Time point	Breastfeeding + Good's coverage	GLMM (Gaussian family + identity link)
Simpson's diversity scores	Group * Time point	Breastfeeding	GLMM (Beta family + logit link)
Bray-curtis dissimilarity matrix T1	Group	Age of infant + Compliance to intervention	PERMANOVA
Bray-curtis dissimilarity matrix T2	Group	Breastfeeding + Compliance to intervention	PERMANOVA
Bray-curtis dissimilarity matrix T3	Group	Sibling	PERMANOVA
Bray-curtis dissimilarity matrix T4	Group		PERMANOVA
Bray-curtis dissimilarity matrix probiotic samples	Time point		Pairwise PERMANOVA
Bray-curtis dissimilarity matrix placebo samples	Time point		Pairwise PERMANOVA

Supplementary Table S10: Neonatal Community State Type Independent Variables (*p* < 0.1)

CST	Independent Variable	Adjusted Odds Ratio (95% CI)	P-value	Placebo samples/ infants	Probiotic samples/ infants
1	Probiotic Group (66%)	5.69 (0.95, 33.42)	**0.057**	15/12	29/24
	Age (one day increase)	1.27 (1.06, 1.52)	**0.009**		
2	-			7/7	5/5
3	Placebo Group (69%)	5.88 (0.85, 500)	**0.071**	9/9	4/4
	Age (one day increase)	0.86 (0.75, 0.98)	**0.028**		
	Breastfeeding (20% increase)	3.23 (1.27, 8.19)	**0.041**		
	Compliance (20% increase)	0.43 (0.22, 0.83)	**0.041**		
	Townsend Score (20% increase)	1.43 (1.00, 2.04)	**0.086**		

Supplementary Table S11: Neonatal Community State Type – Statistical Models and Covariates

Dependent Variable	Minimal Fixed Effects	Additional Significant Fixed Effects	Model
CST 1 (0/1)	Group	Age of infant (days)	GLMM (binomial family + logit link)
CST 2 (0/1)	Group	Age of infant (days)	GLMM (binomial family + logit link)
CST 3 (0/1)	Group	Age of infant (days) + Breastfeeding + Compliance to intervention + Townsend score	GLMM (binomial family + logit link)

Supplementary Table S12: Full list of Taxa for Centrality Measures with Different Keystones between Groups

Time Point	T1		T1		T1		T2		T3		T4		T4		T4		T4	
Centrality Measure (P-value)	Betweenness (0.044)		Closeness (0.002)		Eigenvector (0.002)		Eigenvector (0.026)		Betweenness (0.033)		Degree (0.004)		Betweenness (<0.001)		Closeness (0.004)		Eigenvector (<0.001)	
Group (No. samples/infants)	Placebo (31/25)	Probiotic (31/25)	Placebo (31/25)	Probiotic (31/25)	Placebo (31/25)	Probiotic (31/25)	Placebo (26/25)	Probiotic (26/21)	Placebo (23/22)	Probiotic (23/20)	Placebo (15/13)	Probiotic (15/13)	Placebo (15/13)	Probiotic (15/13)	Placebo (15/13)	Probiotic (15/13)	Placebo (15/13)	Probiotic (15/13)
Network Size (No. Genera)	40	29	40	29	40	29	43	45	40	39	48	57	48	57	48	57	48	57
**Keystones**	Faecalibacterium	Halomonas	Lachnoclostridium	Halomonas	Lachnoclostridium	Bacillus	Flavonifractor	[Ruminococcus] gnavus group	Bacteroides	[Ruminococcus] torques group	Fusicatenibacter	Holdemanella	Coprobacillus	Agathobacter	Coprobacillus	Agathobacter	Lactobacillus	Dorea
	Bradyrhizobium	Bacillus	Enhydrobacter	Bacillus	Enhydrobacter	Halomonas	Blautia	[Ruminococcus] torques group	UBA1819	Bacteroides	Clostridium sensu stricto 1	Agathobacter	Gemella	Staphylococcus	Lactobacillus	Holdemanella	Clostridium sensu stricto 1	Odoribacter
	Lachnoclostridium	Bilophila	Lactobacillus	[Ruminococcus] gnavus group	Bacillus	[Ruminococcus] gnavus group	Raoultella	Erysipelatoclostridium	Intestinibacter	Senegalimassilia	Coprobacillus	Dorea	Dorea	Holdemanella	Lactococcus	Dorea	Coprobacillus	Phascolarctobacterium
	Lactobacillus	Finegoldia	Faecalibacterium	Bilophila	Blautia	Bilophila	Lachnoclostridium	Anaerococcus	Parabacteroides	Staphylococcus	Lactococcus	Odoribacter	Collinsella	Odoribacter	Clostridium sensu stricto 1	Odoribacter	Lacticaseibacillus	Coprococcus
	Enhydrobacter	[Ruminococcus] gnavus group	Veillonella	Finegoldia	Bradyrhizobium	Finegoldia	Peptoniphilus	Citrobacter	Alistipes	Collinsella	Lactobacillus	Phascolarctobacterium	Lactococcus	Collinsella	Collinsella	Phascolarctobacterium	Eggerthella	Holdemanella
	Kosakonia	Erysipelatoclostridium	Ligilactobacillus	Burkholderia-Caballeronia-Paraburkholderia	Lactobacillus	Rothia	Streptococcus	Negativicoccus	Lachnoclostridium	Lacticaseibacillus	Akkermansia	Coprococcus	Clostridium sensu stricto 1	TM7x	Akkermansia	Coprococcus	Lactococcus	Agathobacter
	Halomonas	Parabacteroides	Erysipelatoclostridium	Blautia	Faecalibacterium	Actinomyces	Anaerococcus	Parabacteroides	Erysipelatoclostridium	Parabacteroides	Anaerococcus	Erysipelatoclostridium	Intestinibacter	[Ruminococcus] gnavus group	Peptoniphilus	Staphylococcus	Holdemanella	Varibaculum
	Limosilactobacillus	Streptococcus	Kosakonia	Intestinibacter	Halomonas	Erysipelatoclostridium	[Ruminococcus] torques group	Raoultella	Prevotella	Ruminococcaceae Incertae Sedis	Clostridioides	Klebsiella	Akkermansia	Hungatella	Coprococcus	Collinsella	Negativicoccus	Collinsella
	Ligilactobacillus	Rothia	Bradyrhizobium	Rothia	Veillonella	Parabacteroides	Romboutsia	Hungatella		Cutibacterium	Eggerthella	[Ruminococcus] gnavus group	Lactobacillus	Tyzzerella	Alistipes	TM7x	Atopobium	Clostridioides
	Finegoldia	Eggerthella	Bacillus	Actinomyces	Burkholderia-Caballeronia-Paraburkholderia	Streptococcus	[Clostridium] innocuum group	Lacticaseibacillus		Rothia	Collinsella	[Clostridium] innocuum group	Peptoniphilus	Sutterella	Gemella	[Ruminococcus] gnavus group	Sutterella	Anaerococcus
						Eggerthella	Cutibacterium	Haemophilus		Klebsiella	Alistipes	Sutterella	Coprococcus	Erysipelatoclostridium	Blautia	Sutterella	Peptoniphilus	Ruminococcaceae Incertae Sedis
							Rothia	Enterobacter			Staphylococcus	Tyzzerella	Anaerococcus	Limosilactobacillus	Fusicatenibacter	Erysipelatoclostridium	Alistipes	Terrisporobacter
							Ruminococcaceae Incertae Sedis	Varibaculum			Peptoniphilus	Varibaculum	Clostridioides	Ruminococcaceae Incertae Sedis	Intestinibacter	[Clostridium] innocuum group	Intestinibacter	Staphylococcus
											Sutterella	Collinsella	Staphylococcus	Terrisporobacter	Eggerthella	Terrisporobacter	Akkermansia	Peptoniphilus
											Bilophila	Raoultella	Varibaculum	[Clostridium] innocuum group	Escherichia-Shigella	Ruminococcaceae Incertae Sedis	[Ruminococcus] gnavus group	Flavonifractor
											Intestinibacter	Intestinibacter	Fusicatenibacter	Klebsiella	Dorea	Hungatella	Fusicatenibacter	Sellimonas

Supplementary Table S13: Microbial Network Metrics

Whole Network

Time point	Number of samples/infants		Number of Components		Clustering Co-efficient		Modularity		Edge Density		Natural Connectivity		Average Path Length		Adjusted Rand Index	
	Placebo	Probiotic	Placebo	Probiotic	Placebo	Probiotic	Placebo	Probiotic	Placebo	Probiotic	Placebo	Probiotic	Placebo	Probiotic	Value	P-value
1	31/25	31/25	11	18	0.18	0.14	0.72	0.87	0.04	0.03	0.03	0.03	2.95	1.04	0.06	0.066
2	26/25	26/21	31	8	0.00	0.08	0.79	0.63	0.02	0.04	0.02	0.02	1.38	3.54	0.00	1
3	23/22	23/20	31	10	0.00	0.09	0.74	0.65	0.01	0.04	0.02	0.03	1.37	3.19	-0.02	0.42
4	15/13	15/13	8	11	0.09	0.22	0.57	0.76	0.05	0.03	0.02	0.02	2.71	4.31	0.00	0.913

Largest Connected Component

Time point	Number of samples/infants		LCC size		Relative LCC size		Clustering Co-efficient		Modularity		Edge Density		Natural Connectivity		Average Path Length		Adjusted Rand Index	
	Placebo	Probiotic	Placebo	Probiotic	Placebo	Probiotic	Placebo	Probiotic	Placebo	Probiotic	Placebo	Probiotic	Placebo	Probiotic	Placebo	Probiotic	Value	P-value
1	31/25	31/25	19	6	0.45	0.14	0.15	0.50	0.58	0.17	0.11	0.40	0.07	0.27	3.45	1.35	0.12	0.002
2	26/25	26/21	9	46	0.17	0.87	0.00	0.08	0.33	0.63	0.25	0.05	0.16	0.03	1.70	3.56	0.01	0.377
3	23/22	23/20	6	32	0.13	0.70	0.00	0.09	0.30	0.58	0.33	0.08	0.26	0.04	1.64	3.26	0.02	0.24
4	15/13	15/13	57	44	0.89	0.69	0.09	0.18	0.57	0.69	0.06	0.06	0.02	0.03	2.72	4.43	0.06	0.022

Supplementary Table S14: Abundance of All Antibiotic Resistance Gene Classes Identified in this Study

	PLACEBO GROUP			PROBIOTIC GROUP			PLACEBO vs PROBIOTIC	
	Median abundance:		p-value (within-group comparison):	Median abundance:		p-value (within-group comparison):	p-value (between-group comparison):	
	SP	EP	SP vs EP	SP	EP	SP vs EP	SP	EP
No. samples/infants	8/8	5/5		8/8	13/13			
**Antibiotic class**								
All antibiotic classes	20801.05	14610.85	**0.065**	17550.55	13291.35	0.121	0.798	0.566
Aminocoumarin	1156.44	1033.48	0.622	806.93	439.33	0.121	0.328	0.289
Aminoglycoside	1029.23	740.14	0.724	1304.76	883.15	0.697	0.878	0.633
Beta-lactam	1376.59	824.35	*0.171*	1022.68	460.44	**0.010**	0.645	**0.035**
Carbapenem	110.41	55.66	*0.354*	62.25	59.10	*0.500*	0.878	0.703
Cephalosporin	827.68	448.33	0.524	444.36	201.70	0.121	**0.083**	**0.007**
Cephamycin	41.62	56.55	*0.435*	19.88	43.02	0.140	0.753	0.443
Diaminopyrimidine	214.47	94.44	*0.127*	138.18	36.87	0.336	0.130	0.775
Disinfecting/antiseptic agents	618.95	87.95	**0.011**	678.31	134.43	0.104	0.798	**0.059**
Elfamycin	206.26	208.86	*0.833*	536.86	114.71	**0.003**	0.161	0.336
Fluoroquinolone	2805.04	2164.91	0.622	2956.34	1717.25	**0.076**	0.959	0.443
Fusidane	15.13	9.64	0.354	20.14	4.57	0.210	0.916	1.000
Glycopeptide	3435.81	2442.60	0.284	2776.90	2136.35	0.374	0.645	0.246
Glycylcycline	36.20	33.67	0.524	33.31	22.95	0.500	0.645	0.703
Lincosamide	335.07	198.22	*0.354*	304.46	68.37	0.104	0.798	0.246
Macrolide	3285.32	1755.77	**0.065**	2852.80	1655.09	**0.013**	0.505	1.000
Monobactam	41.83	43.23	0.833	116.04	34.74	0.645	0.528	0.703
Multidrug	9312.27	5949.31	0.222	7162.09	4964.88	**0.008**	0.959	0.703
Mupirocin	107.57	163.76	0.622	811.89	733.96	0.750	**0.059**	**0.095**
Nitroimidazole	141.54	174.86	0.724	388.12	203.17	0.456	0.574	0.289
Nucleoside	301.24	370.76	0.943	399.04	312.04	0.972	0.959	0.924
Penam	190.43	66.87	**0.045**	163.54	46.72	**0.076**	1.000	0.924
Peptide	586.72	292.74	0.171	664.86	558.95	0.860	0.721	0.503
Phenicol	4.32	14.60	0.211	0.00	11.93	**0.063**	0.476	0.924
Phosphonic Acid	268.09	230.47	0.724	397.25	142.55	**0.037**	0.878	0.566
Pleuromutilin	1.44	0.44	0.608	0.34	0.58	0.404	0.365	0.775
Rifamycin	479.96	1217.29	**0.065**	706.82	1524.07	**0.089**	0.161	0.208
Sulfonamide	12.57	14.60	1.000	12.09	10.08	0.456	0.721	1.000
Tetracycline	1408.83	1597.81	1.000	1700.76	1182.29	0.860	0.574	0.703

Supplementary Table S15: MetaCyc pathway IDs

**Pathway Name**	**BioCyc ID**
Anaerobic sucrose degradation	PWY-7345
D-galactose degradation I	PWY-6317
dTDP-L-rhamnose biosynthesis	DTDPRHAMSYN-PWY
Glycogen degradation I	GLYCOCAT-PWY
Heme b biosynthesis from glycine	PWY-5920
L-ascorbate degradation I	PWY0-301
Mannosylglycerate biosynthesis I	PWY-5656
Menaquinol-11 biosynthesis	PWY-5897
Menaquinol-12 biosynthesis	PWY-5898
Menaquinol-13 biosynthesis	PWY-5899
Menaquinol-8 biosynthesis	PWY-5838
Pyruvate fermentation to isobutanol	PWY-7111
Stachyose degradation	PWY-6527
UDP-N-acetyl-D-glucosamine biosynthesis I	UDPNAGSYN-PWY
5-aminoimidazole ribonucleotide biosynthesis	PWY-6277
5-aminoimidazole ribonucleotide biosynthesis I	PWY-6121
5-aminoimidazole ribonucleotide biosynthesis II	PWY-6122
