# Supplementary figures and images for: The Impact of Probiotic Supplementation on the Development of the Infant Gut Microbiota: An Exploratory Follow-Up of a Randomised Controlled Trial
Source: Microorganisms. 2025 Apr 25;13(5):984. doi: 10.3390/microorganisms13050984 (PMC12114409; doi:10.3390/microorganisms13050984)

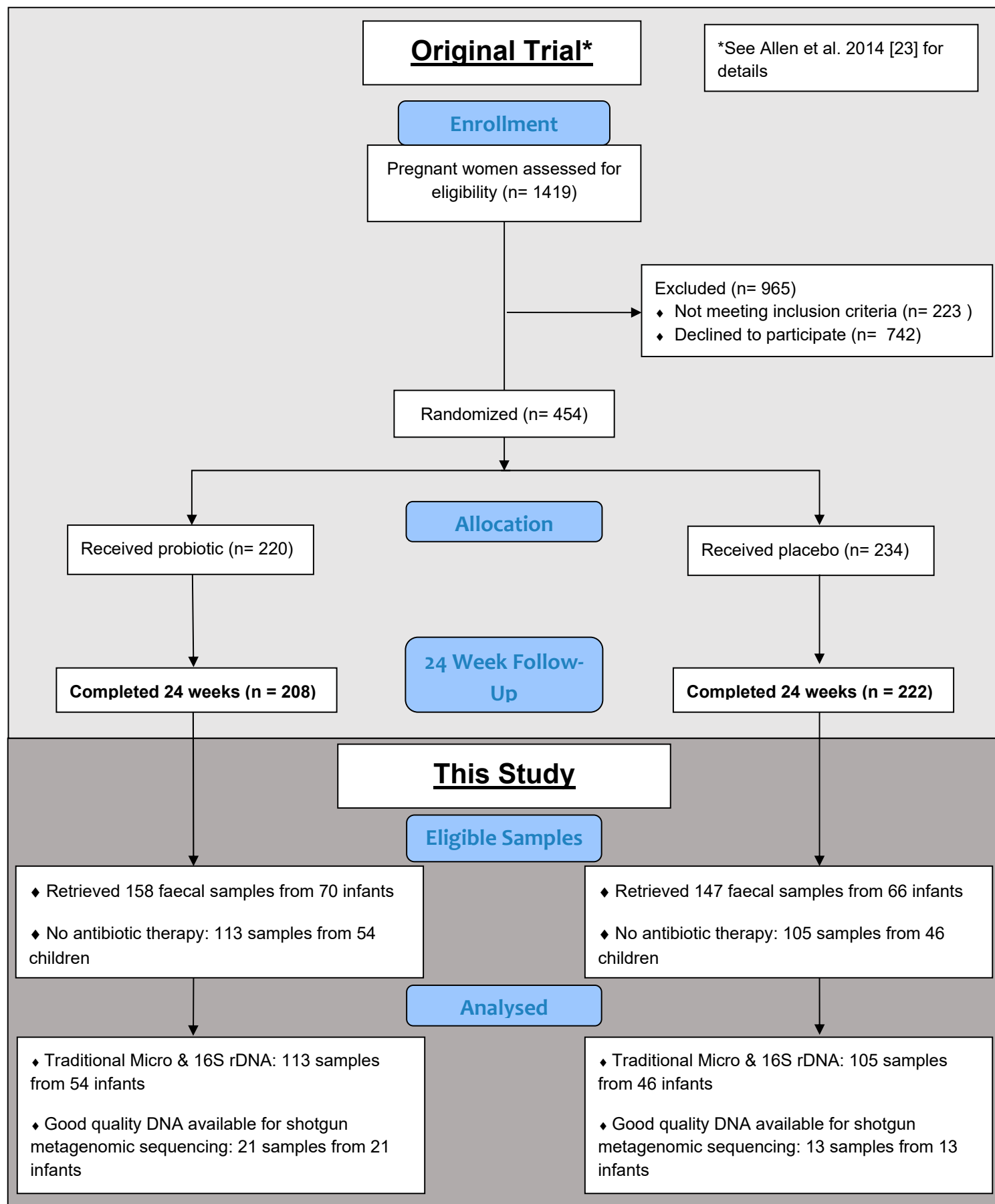

Supplement: Supplementary file 1 [file microorganisms-13-00984-s001.zip › CONSORT Flow Diagram - microorganisms-3570902.pdf]

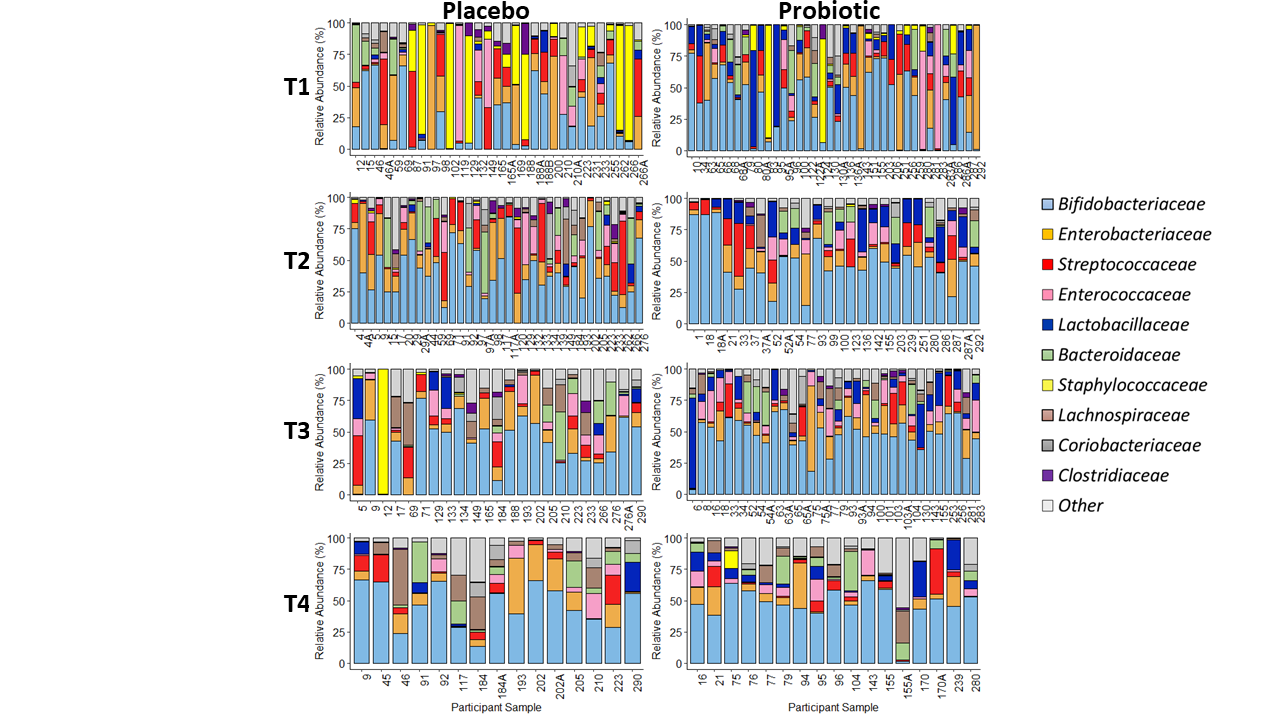

Supplement: Supplementary file 1 [file microorganisms-13-00984-s001.zip › Supplementary Figure S1.tif]

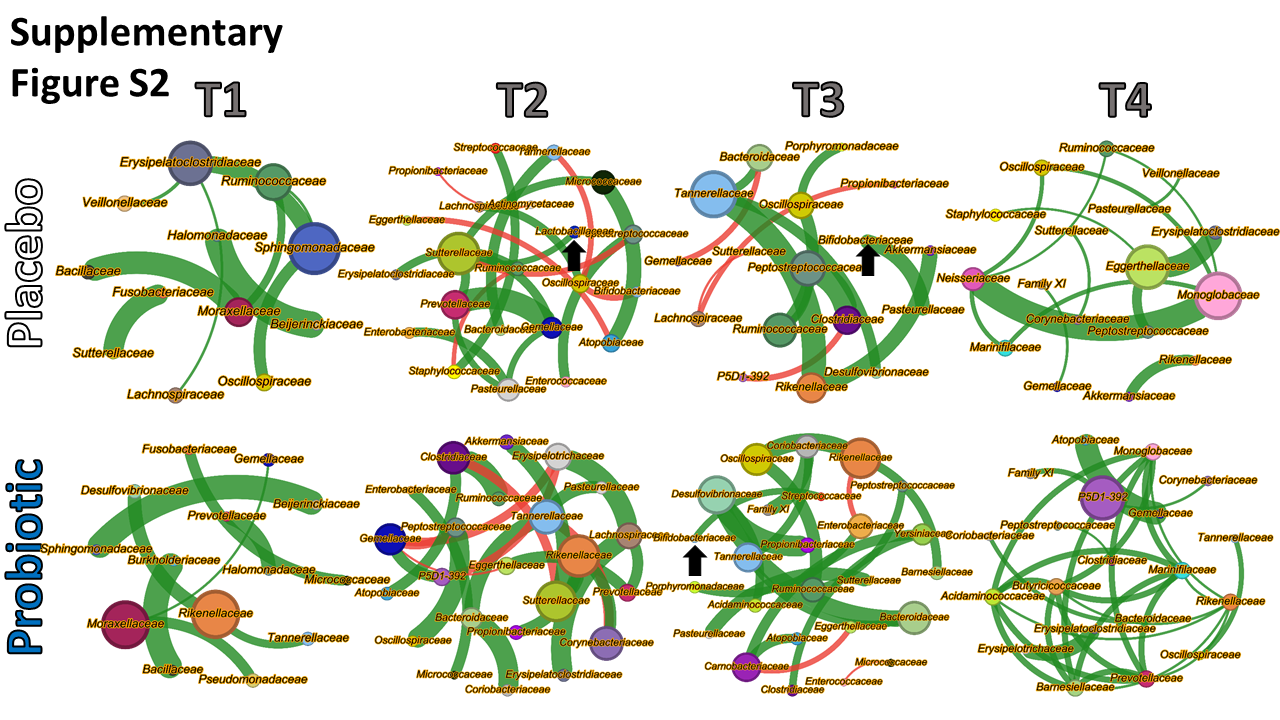

Supplement: Supplementary file 1 [file microorganisms-13-00984-s001.zip › Supplementary Figure S2.tif]
